# ERRATUM

**DOI:** 10.1590/0034-7167.20237603e01

**Published:** 2023-10-30

In the article “Perceptions of adults with obesity about multiprofessional remote monitoring at the beginning of the COVID-19 pandemic”, with DOI number: https://doi.org/10.1590/0034-7167-2020-0710, published in Revista Brasileira de Enfermagem, 2021;74(Suppl 1): e20200710, on page 8:

Where it read:

17. Bim H, Gonçalves ECA, Bolognese MA, Westpal G, Thon RA, Castilho MM, et al. Perfil de pacientes que procuram um programa multiprofissional de tratamento da obesidade[Internet]. 2018 [cited 2020 Jun 19]. Available from: http://www.revistaagape.com.br/index.php/revistaagape/article/view/9

It reads:

17. Bim H, Gonçalves ECA, Bolognese MA, Westpal G, Thon RA, Castilho MM, et al. Perfil de pacientes que procuram um programa multiprofissional de tratamento da obesidade[Internet]. 2018 [cited 2020 Jun 19]. Available from: https://periodicos.uem.br/ojs/index.php/healtheduc/article/view/64450

